# Basal ganglia dysfunction in idiopathic REM sleep behaviour disorder parallels that in early Parkinson’s disease

**DOI:** 10.1093/brain/aww124

**Published:** 2016-06-12

**Authors:** Michal Rolinski, Ludovica Griffanti, Paola Piccini, Andreas A. Roussakis, Konrad Szewczyk-Krolikowski, Ricarda A. Menke, Timothy Quinnell, Zenobia Zaiwalla, Johannes C. Klein, Clare E. Mackay, Michele T. M. Hu

**Affiliations:** ^1^ 1 Oxford Parkinson’s Disease Centre (OPDC), Oxford, UK; ^2^ 2 Nuffield Department of Clinical Neurosciences, University of Oxford, Oxford, UK; ^3^ 3 Centre for the functional MRI of the Brain (FMRIB), University of Oxford, Oxford, UK; ^4^ 4 Division of Clinical Neurosciences and MRC Clinical Sciences Centre, Faculty of Medicine, Hammersmith Hospital, Imperial College London, London, UK; ^5^ 5 Respiratory Support and Sleep Centre, Papworth Hospital, Cambridge, UK; ^6^ 6 Department of Clinical Neurophysiology, John Radcliffe Hospital, Oxford, UK; ^7^ 7 Department of Psychiatry, University of Oxford, Oxford, UK

**Keywords:** Parkinson’s disease, imaging, rapid eye movement sleep behaviour disorder

## Abstract

**See Postuma (doi:10.1093/aww131) for a scientific commentary on this article.**

Resting state functional magnetic resonance imaging dysfunction within the basal ganglia network is a feature of early Parkinson’s disease and may be a diagnostic biomarker of basal ganglia dysfunction. Currently, it is unclear whether these changes are present in so-called idiopathic rapid eye movement sleep behaviour disorder, a condition associated with a high rate of future conversion to Parkinson’s disease. In this study, we explore the utility of resting state functional magnetic resonance imaging to detect basal ganglia network dysfunction in rapid eye movement sleep behaviour disorder. We compare these data to a set of healthy control subjects, and to a set of patients with established early Parkinson’s disease. Furthermore, we explore the relationship between resting state functional magnetic resonance imaging basal ganglia network dysfunction and loss of dopaminergic neurons assessed with dopamine transporter single photon emission computerized tomography, and perform morphometric analyses to assess grey matter loss. Twenty-six patients with polysomnographically-established rapid eye movement sleep behaviour disorder, 48 patients with Parkinson’s disease and 23 healthy control subjects were included in this study. Resting state networks were isolated from task-free functional magnetic resonance imaging data using dual regression with a template derived from a separate cohort of 80 elderly healthy control participants. Resting state functional magnetic resonance imaging parameter estimates were extracted from the study subjects in the basal ganglia network. In addition, eight patients with rapid eye movement sleep behaviour disorder, 10 with Parkinson’s disease and 10 control subjects received ^123^I-ioflupane single photon emission computerized tomography. We tested for reduction of basal ganglia network connectivity, and for loss of tracer uptake in rapid eye movement sleep behaviour disorder and Parkinson’s disease relative to each other and to controls. Connectivity measures of basal ganglia network dysfunction differentiated both rapid eye movement sleep behaviour disorder and Parkinson’s disease from controls with high sensitivity (96%) and specificity (74% for rapid eye movement sleep behaviour disorder, 78% for Parkinson’s disease), indicating its potential as an indicator of early basal ganglia dysfunction. Rapid eye movement sleep behaviour disorder was indistinguishable from Parkinson’s disease on resting state functional magnetic resonance imaging despite obvious differences on dopamine transported single photon emission computerized tomography. Basal ganglia connectivity is a promising biomarker for the detection of early basal ganglia network dysfunction, and may help to identify patients at risk of developing Parkinson’s disease in the future. Future risk stratification using a polymodal approach could combine basal ganglia network connectivity with clinical and other imaging measures, with important implications for future neuroprotective trials in rapid eye movement sleep behaviour disorder.

## Teaser

Idiopathic REM sleep behaviour disorder (RBD) is associated with frequent conversion to Parkinson’s disease. Rolinski *et al.* show that resting-state fMRI differentiates cases of RBD and Parkinson’s disease from controls with high sensitivity (96%) and specificity (74–78%). Basal ganglia network connectivity may reveal future Parkinson’s disease before motor symptom onset.

## Graphical abstract

**Figure:**
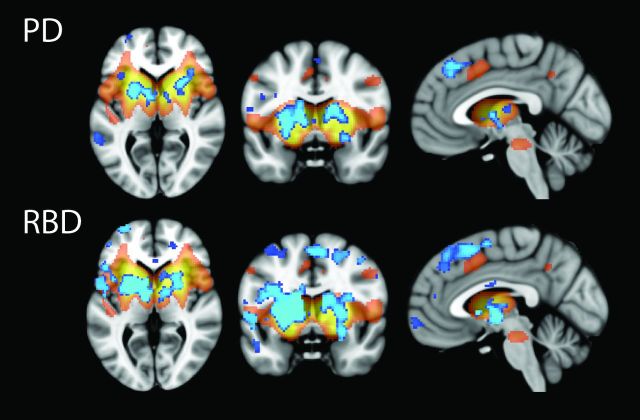
**See Postuma (doi:10.1093/aww131) for a scientific commentary on this article.** Idiopathic REM sleep behaviour disorder (RBD) is associated with frequent conversion to Parkinson’s disease. Rolinski *et al.* show that resting-state fMRI differentiates cases of RBD and Parkinson’s disease from controls with high sensitivity (96%) and specificity (74–78%). Basal ganglia network connectivity may reveal future Parkinson’s disease before motor symptom onset.

## Introduction

Significant abnormalities in resting state functional MRI have previously been reported by our group within the basal ganglia network (BGN) of patients with early Parkinson’s disease (Szewczyk-Krolikowski *et al.*, 2014*a*; Rolinski *et al.*, 2015). While this approach shows promise as a diagnostic biomarker in the early motor phases of Parkinson’s disease, it is unclear whether these changes are present in prodromal disease.

Over the past 20 years, increasing evidence has emerged for idiopathic rapid eye movement (REM) sleep behaviour disorder (RBD), occurring in the absence of any other clinically defined neurological disorder, being associated with the prodromal stages of a number of neurodegenerative conditions, predominantly Parkinson’s disease (Schenck *et al.*, 1996, 2013; Iranzo *et al.*, 2006; Postuma *et al.*, 2009*a*, *b*; Boot *et al.*, 2012; Wing *et al.*, 2012). Therefore, RBD may be considered as the strongest predictor of neurodegeneration available by far (Postuma *et al.*, 2010), with many RBD patients showing early features of neurodegenerative conditions (Fantini *et al.*, 2006; Postuma *et al.*, 2006, 2009*a*, *b*). Cheap, safe and reliable means of identifying those at highest risk of developing Parkinson’s disease would facilitate the targeted use of novel disease-modifying therapies and revolutionize clinical trials in this field.

In this study, we set out to explore the potential of resting state functional MRI to quantify basal ganglia dysfunction in patients with RBD. Moreover, postulating that in most cases (Schenck *et al.*, 1996, 2013; Iranzo *et al.*, 2006; Postuma *et al.*, 2009*a*, *b*; Boot *et al.*, 2012; Wing *et al.*, 2012), RBD represents the prodromal stages of Parkinson’s disease, we endeavoured to draw direct comparisons with patients with established, clinically defined, Parkinson’s disease. Hence, we strived to assess the hypothesis that resting state functional MRI signature of Parkinson’s exists before the motor disease can be diagnosed. For comparison, we analysed ^123^I-ioflupane uptake in a subset of patients, an established surrogate of dopaminergic decline.

## Materials and methods

### Subjects

#### MRI

The study was undertaken with the understanding and written consent of each subject, with the approval of the local NHS committee, and in compliance with national legislation and the Declaration of Helsinki.

Twenty-six patients with RBD (22 males, age 67.0 ± 7.7 years, symptom duration 7.0 ± 3.6 years, disease duration 2.4 ± 2.1 years) were consecutively recruited from the sleep disorders clinics at the John Radcliffe Hospital, Oxford and Papworth Hospital, Cambridge. The diagnosis of RBD was made on the basis of polysomnographic evidence according to standard International Classification of Sleep Disorders-II criteria by a consultant specializing in sleep disorders (Lapierre and Montplaisir, 1992). RBD was defined as an increase in tonic or phasic chin EMG activity during REM sleep and, either history of elaborate motor activity associated with dream content, or the presence of behavioural manifestations occurring during REM sleep during polysomnographic recordings (Lapierre and Montplaisir, 1992). Patients were excluded if RBD was judged by their clinical team to be secondary to medication use, or was associated with other neurological conditions, including narcolepsy, Parkinson’s disease, dementia or multiple system atrophy. RBD symptom duration was calculated as the time from the patient’s defined symptom onset; RBD diagnosis duration was taken from the date of the diagnostic polysomnogram.

Forty-eight age- and gender-matched patients with a clinical diagnosis of idiopathic Parkinson’s disease according to the UK Parkinson’s disease Society Brain Bank criteria (Hughes *et al.*, 1992) [31 males, age 67.0 ± 7.7 years, disease duration 1.8 ± 1.5 years, Unified Parkinson’s Disease Rating Scale (UPDRS) III 26.4 ± 12.3, Hoehn and Yahr 1–2] and 23 healthy control subjects were recruited from the Oxford Parkinson’s Disease Centre patient cohort (Rolinski *et al.*, 2014). Further clinical characteristics across the RBD, Parkinson’s disease and control groups are summarized in Table 1, and were compared using Kruskal-Wallis test with a *post hoc* Dunn’s test. Twenty-eight patients with Parkinson’s disease and 11 healthy control subjects overlapped with those included in our previous study (Szewczyk-Krolikowski *et al.*, 2014*a*). Patients ON dopaminergic medications were scanned after at least a 12 h withdrawal, in a clinically defined ‘OFF’ state. The control subjects had no evidence of significant neurological or psychiatric illness during structured interview and formal neurological examination with a trained movement disorders neurologist [M.R./K.S.K., see Szewczyk-Krolikowski *et al.* (2014*b*) for full protocol details].

**Table 1:** Comparison of clinical characteristics in RBD, Parkinson’s disease and control groups

Variable	RBD (*n* = 26)	Parkinson’s disease	Controls (*n* = 23)	*P*-value^a^	*P*-value^b^	*P*-value^b^
		(*n* = 48)		RBD versus PD versus Controls	RBD versus PD	RBD versus Controls
UPDRS III	3.3 (3.5)	26.4(12.3)	0.7 (1.1)	<0.001	<0.001	0.067
BDI	9.1 (8.6)	7.7 (4.6)	4.9 (5.6)	0.035	0.40	0.020
Leeds Depression	3.9 (3.6)	3.7 (3.0)	2.9 (3.0)	0.47	0.44	0.17
Leeds Anxiety	2.9 (2.3)	2.6 (2.4)	1.9 (2.7)	0.12	0.27	0.022
MoCA^c^	25.3 (2.9)	27.4 (2.3)	28.2 (1.4)	<0.001	<0.001	<0.001
MMSE	27.3 (1.7)	28.5 (1.5)	29.3 (1.0)	<0.001	<0.001	<0.001
Phonemic fluency^d^	10.9 (4.7)	12.9 (3.8)	15.0 (3.0)	0.006	0.046	<0.001
Semantic fluency^d^	9.8 (3.1)	11.3 (2.9)	13.2 (3.0)	0.003	0.048	<0.001

PD = Parkinson’s disease; BDI = Becks Depression Inventory; MoCA = Montreal Cognitive Assessment; MMSE = Mini-Mental State Examination

^a^Kruskal-Wallis.

^b^Dunn’s test for pairwise comparisons.

^c^Adjusted for education years.

^d^Fluencies are age adjusted.

Data shown are mean (SD).

#### SPECT

Eight RBD patients had one single single photon emission computerized tomography (SPECT) scan with ^123^I-ioflupane (six males; age 68.5 ± 6.8; disease duration from diagnosis 5.3 ± 3.0; disease duration from onset; 6.3 ± 3.2, Table 3). For one RBD patient from this subgroup, MRI data were unavailable for technical reasons. Ten separately recruited age- and sex-matched patients with a clinical diagnosis of idiopathic Parkinson’s disease according to the UK Parkinson’s disease Society Brain Bank criteria (six males, age 68.6 ± 6.1; disease duration from diagnosis 0.4 ± 0.6; disease duration from onset; 1.5 ± 0.6) had a SPECT scan with ^123^I-ioflupane similarly to the group of RBDs. All Parkinson’s disease patients who undertook SPECT scan with ^123^I-ioflupane had early unilateral disease (Hoehn and Yahr = 1.0). In addition, a group of 10 separately recruited healthy volunteers (five males, 60.5 ± 8.9) were recruited as healthy controls. All participants of the SPECT arm of the study were not taking any dopaminergic or serotonergic medication.

**Table 2:** Regions showing significantly lower basal ganglia network activity in patients with Parkinson’s disease and RBD, compared to healthy controls

Cluster location	Cluster size (voxels)	Most significant voxel (MNI coordinates: *x*, *y*, *z*)
**Parkinson’s disease**		
L putamen	1583	−24, 4, 0
R paracingulate gyrus	1493	4, 26, 42
R putamen	1127	24, 12, 8
L inferior temporal gyrus	324	−58, −52, −12
R putamen	216	28, 0, −10
L inferior frontal gyrus	133	−50, 10, 12
L frontal pole	105	48, 26, 28
**RBD**		
L putamen (extending into R putamen)	11 639	−24, 6, 0
R frontal orbital cortex	703	50, 28, −12
L frontal orbital cortex	455	−26, 18, −12
R middle frontal gyrus	133	42, 16, 36
R cingulate gyrus	66	16, −38, 32
L middle temporal gyrus	36	−54, −16, −16
L middle temporal gyrus	16	−62, −46, 0

L = left; R = right.

*P* < 0.05 FWE corrected, cluster ≥10 voxels.

**Table 3:** Clinical characteristics of SPECT participants

	RBD patients	Healthy controls	Parkinson’s disease patients
Number of subjects	8	10	10
Sex ratio (male:female)	6M:2F	5M:5F	6M:4F
Age at the time of the scan (years)	68.5 ± 6.80	60.5 ± 8.90	68.6 ± 6.10
MMSE score	28.4 ± 1.30	29.7 ± 0.67	28.5 ± 1.08
Hoehn and Yahr stage	n/a	n/a	1 ± 0
Disease duration from onset (years)	6.3 ± 3.20	n/a	1.5 ± 0.62
Disease duration from diagnosis (years)	5.3 ± 3.01	n/a	0.4 ± 0.59

Data represent mean ± 1 SD.

MMSE = Mini-Mental State Examination; n/a = not applicable

### Data acquisition

#### MRI

Data acquisition was performed at the Oxford Centre for Clinical Magnetic Resonance Research (OCMR) using a 3 T Trio Siemens MRI scanner equipped with a 12-channel coil.

T_1_-weighted images were obtained using a 3D magnetization prepared-rapid acquisition gradient echo (MPRAGE) sequence (192 axial slices, flip angle 8°, 1 × 1 × 1 mm^3^ voxel size, echo time/repetition time/inversion time = 4.7 ms/2040 ms/900 ms) for volumetric and registration purposes.

Resting state functional MRI was acquired using gradient echo planar imaging (EPI) (repetition time = 2000 ms, echo time = 28 ms, flip angle = 89°, resolution = 3 × 3 × 3.5 mm). Thirty-four axial slices were acquired per volume, covering both hemispheres with incomplete coverage of the cerebellum; 180 repetitions were acquired in 6 min. Participants were instructed to remain still and awake with their eyes open.

#### SPECT

Prior to the administration of ^123^I-ioflupane, thyroid gland blockade was performed by oral administration of potassium iodide 60 mg twice daily starting 24 h prior to the SPECT scan day, and for three consecutive days in total, in accordance with the clinical protocol of Imperial College Healthcare NHS Trust’s Nuclear Medicine Department. SPECT data acquisition was performed at the Charing Cross Hospital, using a Symbia™ SPECT–CT scanner (Siemens). Patients were scanned in a supine position using dedicated head restraint to minimize movement.

SPECT images were acquired 3 h after intravenous bolus injection of ^123^I-ioflupane. SPECT images were obtained continuously while participants were at rest for ∼45 min (acquisition parameters: 128 views with 128 × 128 matrix and 1.45 zoom with 30 s per view in step-and-shoot mode; 15% energy window centred on the 159 keV photopeak of ^123^I; 2 million total counts). The mean activity dose of ^123^I-ioflupane was 185 MBq (provided as DaTscan™ injection, GE Healthcare). Tomographic imaging data were reconstructed using the OSEM algorithm incorporating corrections for attenuation, scatter and resolution using Hybrid Recon™ software (HERMES Medical Solutions, Sweden). Reconstructed images were smoothed using a 3D Gaussian filter (full-width at half-maximum = 0.70 cm). SPECT imaging of patients with RBD was performed within 8 ± 5.6 months apart from magnetic resonance scanning.

### Data analysis

#### MRI

Analyses were performed using tools from the FMRIB Software Library (FSL) (Jenkinson *et al.*, 2012). Voxel-based morphometry analyses of the T_1_-MPRAGE data were carried out using FSL-VBM (Douaud *et al.*, 2009), testing for reduction of grey matter concentrations in Parkinson’s disease and RBD patients compared to controls. We used the recommended FSL pipeline, including segmentation with FAST, non-linear registration with FNIRT and construction of a study-specific standard space template.

Resting state analysis was performed using probabilistic independent component analysis (ICA) as implemented in the Multivariate Exploratory Linear Optimized Decomposition into Independent Component FSL tool (MELODIC) (Beckmann and Smith, 2004). Individual pre-statistical processing consisted of motion correction, brain extraction, unwarping using fieldmap data, spatial smoothing using Gaussian kernel of full-width at half-maximum of 6 mm, and high-pass temporal filtering of 150 s. To account for the effect of motion, non-neural physiology, scanner artefacts and other confounds, we used FIX, an ICA-based denoising approach (Griffanti *et al.*, 2014; Salimi-Khorshidi *et al.*, 2014). Once preprocessed, data were linearly registered to the corresponding structural image using FLIRT (Jenkinson *et al.*, 2002), and registered to Montreal Neurological Institute (MNI) space using non-linear registration.

A previously developed template of resting state networks generated from 80 healthy elderly participants was used (Szewczyk-Krolikowski *et al.*, 2014*a*). It included the BGN and 21 residual noise components that were not fully removed by FIX and were identified as residual noise based on the identification of standard noise components (Beckmann, 2012) and location of signal peaks in non-grey matter areas (e.g. white matter, CSF, skull), were also included as nuisance covariates. The dual regression approach (Filippini *et al.*, 2009) was used to identify individual temporal dynamics and the associated spatial maps of the resting state networks.

Statistical comparisons were performed using permutation-based non-parametric inference within the framework of the GLM using Randomise (v2.1). Results were considered significant for *P* < 0.05, after correction for multiple comparisons (family-wise error) using the threshold-free cluster enhancement (TFCE) approach (Smith and Nichols, 2009), which enhances sensitivity to spatially distributed effects. The design included linear regressors for age and sex.

A *post hoc* analysis was performed to further characterize the connectivity changes within the BGN between the study groups. For each participant, parameter estimates representing the connectivity of a given voxels with the time-course of the whole network, were averaged within a binary mask containing only significant clusters from the voxel-wise analysis. A receiver operating characteristic (ROC) curve was generated to assess the separation between the two groups. Last, to assess the intra-network connectivity within individual parts of the basal ganglia, subcortical masks were created from the Harvard-Oxford Subcortical Atlas (Mazziotta *et al.*, 2001). The generated masks were used to mean parameter estimates from subject-specific BGN spatial maps, from the following regions of interest: caudate, pallidum and the posterior and anterior putamen, bilaterally. The boundary between the anterior and posterior putamen was taken to be the posterior aspect of the fornix on the axial plane.

#### SPECT

^123^I-ioflupane SPECT data were analysed using the BRASS software (HERMES medical solutions, Sweden) following a semi-quantitative approach. Each individual’s reconstructed image was automatically registered to a predefined template, provided with the software. Following automatic alignment, all scans were inspected visually and manually to fit to the predefined template where necessary. Uptake ratios of ^123^I-ioflupane were calculated for each striatum, caudate, putamen, anterior and posterior putamen relative to the non-specific uptake measured in the occipital cortex. The uptake is defined as the specific binding ratio [(striatal counts–background counts)/background counts]. The specific DAT binding as reflected by ^123^I-ioflupane uptake values was calculated for both hemispheres. The average binding for region of interest was calculated per individual as the mean uptake value for both hemispheres.

We tested for differences in tracer uptake between Parkinson’s disease, RBD and control groups using the Kruskal-Wallis test. *Post hoc* Dunn’s tests were performed to identify differences between (i) Parkinson’s disease and controls; (ii) Parkinson’s disease and RBD; and (iii) RBD and controls. All tests used a threshold of *P* < 0.05 one-tailed. Applying methodology similar to that used in the Parkinson Associated Risk Syndrome Study (Jennings *et al.*, 2014), we determined the percentage of expected ^123^I-ioflupane tracer uptake in the lowest putamen of each RBD and Parkinson’s individual by comparing to the mean of the lowest putamen in the 10 control subjects. Individual subjects were categorized as having dopamine transporter (DaT) deficit (≤65% expected lowest putamen ^123^I-ioflupane binding), intermediate (65–80% expected lowest putamen ^123^I-ioflupane binding), or no DaT deficit (>80% expected lowest putamen ^123^I-ioflupane binding).

### Correlation analysis: MRI and SPECT

We tested for significant correlation between regional ^123^I-ioflupane tracer uptake, and BGN parameter estimates for the whole BGN network, and for the individual regions studied, that is caudate nucleus, whole putamen, anterior and posterior putamen, using Spearman’s rank correlation.

Due to the low number of subjects receiving SPECT and the exploratory nature of the DAT analysis, we did not apply correction for multiple comparisons.

## Results

### Voxel-based morphometry

Voxel-based morphometry analysis did not yield any significant grey matter differences between the three groups, including within cortical or brainstem subregions. Hence, voxel-wise grey matter masks were not included as covariates in the functional MRI analysis.

### Resting state network analysis

The mean relative (time point-to-time point) and absolute head motion during functional MRI acquisition did not differ significantly between the three groups [*F*(2,94) = 2.93, *P* = 0.06 and *F*(2,94) = 1.58, *P* = 0.2, respectively].

**Figure 1:**
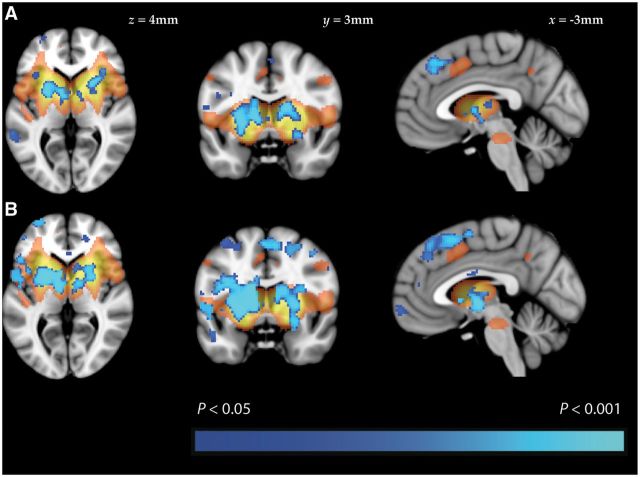
**Results of resting state functional MRI analysis.** Group difference maps illustrate clusters of significantly reduced connectivity (blue) in patients with (**A**) Parkinson’s disease and (**B**) RBD, when compared to healthy controls. Clusters are thresholded at *P* < 0.05 after TFCE correction. A map of the BGN in shown in orange (thresholded at Z < 2.6).

Significantly reduced coactivation within the BGN was found in patients with Parkinson’s disease and RBD, when compared to healthy controls (Fig. 1). In both cases, significant clusters were found within the basal ganglia, as well as frontal regions, such as the cingulate and paracingulate gyri, the frontal orbital cortices and the inferior and middle frontal gyri (Table 2). Voxel-wise comparison did not reveal any statistically significant differences when patients with RBD were compared to patients with established Parkinson’s disease.

**Figure 2:**
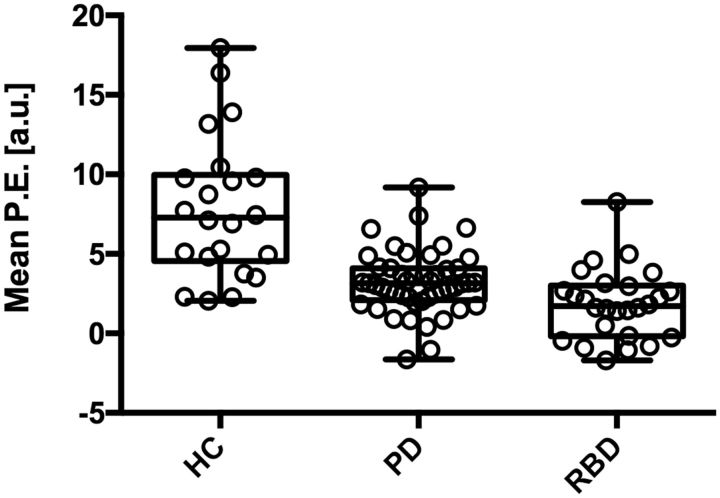
**Mean parameter estimates extracted from significant clusters that appeared in both the healthy controls versus Parkinson’s disease and healthy controls versus RBD comparisons.** Each boxplot represents (from *bottom* to *top*) quartile 1, median, and quartile 3, with whiskers representing the minimum and maximum mean parameter estimate (P.E.) values for the group.

Individual mean parameter estimates were extracted from the significant clusters. In the case of Parkinson’s disease, the mean parameter estimate differentiated the disease group from the healthy controls with a sensitivity and specificity of 95.8% [95% confidence interval (CI) 85.6–99.5] and 73.9% (95% CI 51.6–89.8), respectively. The area under the curve (AUC) was 0.90 (95% CI 0.83–0.98). The RBD cases could be differentiated from the healthy controls with a sensitivity of 96.2 (95% CI 80.4–99.9) and specificity of 78.3 (95% CI 56.3–92.5). The AUC was 0.92 (95% CI 0.85–1.00). The distribution of individual mean parameter estimates extracted from the clusters that showed significant difference in both comparisons is illustrated in Fig. 2.

To control for laterality we compared the parameter estimates extracted from the BGN within the areas that showed significant differences between Parkinson’s disease and controls (i) between Parkinson’s disease subjects with unilateral versus bilateral signs on the UPDRS III; and (ii) between Parkinson’s disease subjects with a higher UPDRS III scores for the left side and Parkinson’s disease subjects with higher UPDRS III scores for the right side. No significant differences were found in either case. To further investigate the influence of laterality of symptoms with functional connectivity we correlated the parameter estimates extracted from the BGN with the contralateral UPDRS III score. No significant correlation was found.

### Anatomical regions of interest

**Figure 3:**
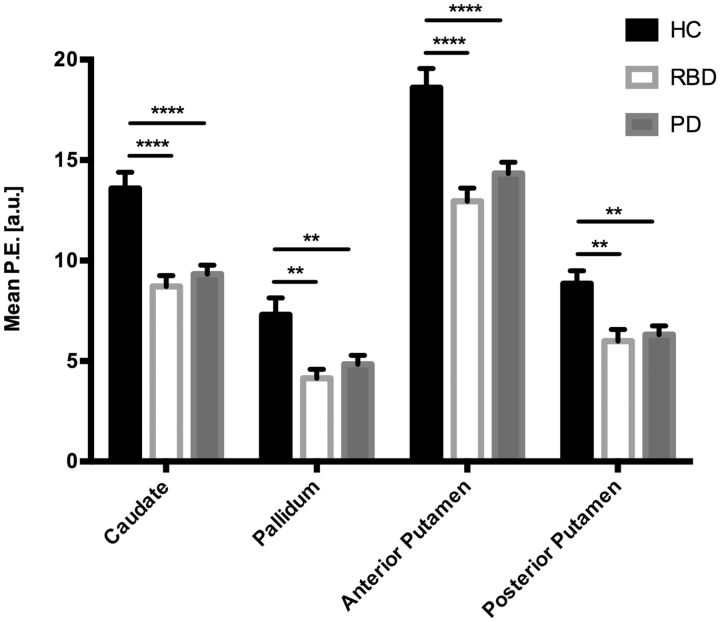
**Mean parameter estimates extracted from anatomical regions.** The mean parameter estimate (P.E.) values were significantly lower in both the Parkinson’s disease and RBD groups, when compared to the healthy control group, in all four areas tested. There was no significant difference in any of the regions when RBD patients were compared to those with established Parkinson’s disease. The bars represent the group mean and the standard error of the mean. *P*-values corrected using Dunnett’s multiple comparison test. *P < 0.05; ^**^P < 0.01; ^***^P < 0.001; ^****^P < 0.0001.

The mean parameter estimates extracted from anatomical regions within the basal ganglia are shown in Fig. 3. Both the Parkinson’s disease and RBD groups had significantly lower parameter estimate values within the caudate, pallidum, and the anterior and posterior putamen, when compared to the healthy control group. There were no statistically significant differences between the RBD and Parkinson’s disease groups.

### SPECT data

The clinical characteristics and mean uptake values from of the ^123^I-ioflupane SPECT study are summarized in Tables 3 and 4.

**Table 4:** Uptake values of ^123^I-ioflupane SPECT

	RBD patients	Healthy controls	Parkinson’s disease patients
Striatum	2.93 ± 0.45	3.26 ± 0.30	2.15 ± 0.52^***,†^
Caudate	3.19 ± 0.70	3.43 ± 0.43	2.47 ± 0.53^**,†^
Putamen	2.69 ± 0.39	3.10 ± 0.29	1.86 ± 0.54^***,†^
Anterior putamen	3.03 ± 0.46	3.50 ± 0.33	2.20 ± 0.63^***^
Posterior putamen	2.32 ± 0.44	2.67 ± 0.32	1.30 ± 0.44^***,†^

Data represent mean ± 1 SD.

*P < 0.05, ^**^P < 0.01, ^***^P < 0.001. Comparison to *controls, or ^†^RBD at *P* < 0.05

**Figure 4:**
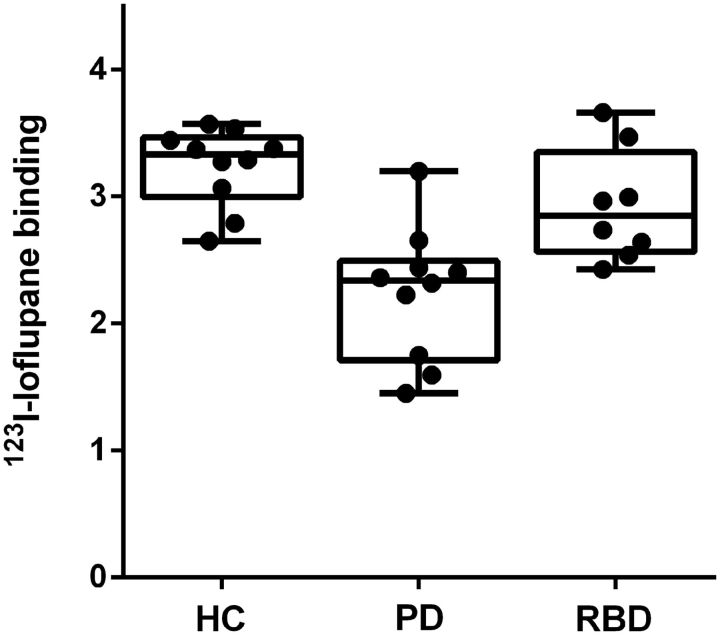
**^123^I–ioflupane binding in the lowest putamen of control, Parkinson’s disease and RBD subjects.** Each boxplot represents (from *bottom* to *top*) quartile 1, median, and quartile 3, with whiskers representing the minimum and maximum ^123^I–ioflupane binding for the group. HC = healthy controls; PD = Parkinson’s disease.

Parkinson’s disease patients showed reduced ^123^I-ioflupane uptake in all five regions of interest compared to control subjects (*P* < 0.01). RBD patients showed a trend towards reduced ^123^I-ioflupane uptake compared to normal controls that failed to reach significance in all five regions of interest. Finally, Parkinson’s disease patients showed reduced ^123^I-ioflupane uptake compared to RBD patients in the striatum (*P* < 0.05), caudate (*P* < 0.05), putamen (*P* < 0.05), and posterior putamen (*P* < 0.05). Figure 4 shows individual level ^123^I-ioflupane DaT binding in the putamen with the lowest uptake (right or left) for healthy controls, Parkinson’s disease and RBD subjects. Eight of ten Parkinson’s disease subjects and 1 out of 8 RBD subjects were categorized as having DaT deficit (≤65% expected lowest putamen ^123^I-ioflupane binding), with 1 of 10 Parkinson’s disease and two of eight RBD subjects categorized as having intermediate DaT deficit (65–80% expected lowest putamen ^123^I-ioflupane binding). The mean uptake value of ^123^I-ioflupane for the RBD group in the putamen was 13.2% lower than the mean value of the normal controls, and 30.8% higher than the mean value of the Parkinson’s disease patients.

### Correlation analysis: MRI and SPECT

Both MRI and SPECT data were available for seven of eight subjects. We did not detect correlation between regional ^123^I-ioflupane tracer uptake, and BGN parameter estimates for any of the striatal subregions, or the striatum as a whole.

## Discussion

In this study, we explore the potential of resting state functional MRI to quantify basal ganglia dysfunction in patients with RBD and to identify an imaging signature of Parkinson’s disease before the onset of motor disease. To this end, we performed voxel-wise and region of interest analyses of the BGN, directly comparing the results to healthy controls and patients with established, clinically defined, Parkinson’s disease. Additionally, we explore the relationship of basal ganglia dysfunction quantified with resting state functional MRI with dopaminergic state as assessed by ^123^I-ioflupane SPECT.

Our data show that widespread aberrant connectivity within the BGN is detectable using resting state functional MRI in patients with RBD who do not manifest significant motor impairment. These changes are most prominent within the basal ganglia themselves, with further extra-striatal changes observed predominantly in the frontal lobes. Moreover, having replicated our previous results in early Parkinson’s disease (Szewczyk-Krolikowski *et al.*, 2014*a*) in a larger group, we show that BGN connectivity in patients with RBD directly mirrors that observed in established Parkinson’s disease, that is, the Parkinson’s disease and RBD groups show a comparable decline in BGN function relative to controls.

Dopaminergic transmission, however, differs between these two groups. In keeping with previously reported data (Eidelberg *et al.*, 1990), our SPECT analysis demonstrated an intermediate dopaminergic phenotype in some RBD patients. While reduction of dopaminergic terminals in the striatum failed to reach significance at a group level in RBD relative to controls, three of eight RBD compared to 9 of 10 Parkinson’s disease subjects were categorized as having dopaminergic deficits based on putamen ^123^I-ioflupane uptake.

Our results fit with the hypothesis that, in many, RBD may represent the prodromal stage of Parkinson’s disease, with an estimated period of 10–15 years of progressive neuronal loss before the onset of the core motor symptoms (Hawkes, 2008). In line with evidence from radiotracer imaging studies, we found significantly decreased functional connectivity affecting the caudate, putamen and globus pallidus, bilaterally. Previous SPECT scans have demonstrated decreased ^123^I-FP-CIT uptake in the striatum of patients with idiopathic RBD, with ∼40% of patients classified as having a clinically abnormal scan (Eisensehr *et al.*, 2003; Iranzo *et al.*, 2010). Similarly, decreased ^11^C-dihydrotetrabenazine (^11^C-DTBZ) striatal binding on PET scanning suggests loss of dopaminergic neurons in patients with RBD (Albin *et al.*, 2000). Supporting the concept of a BGN dysfunction in RBD, a previous PET study has established an expression of the metabolic Parkinson disease-related spatial covariance pattern in RBD using ^18^FDG-PET (Holtbernd *et al.*, 2014).

In the only previously published study of resting state connectivity in RBD, Ellmore and colleagues (2013) reported on seed-based nigrostriatal and nigrocortical connectivity in 10 RBD patients, 11 Parkinson’s disease patients and 10 healthy controls. The authors reported altered connectivity between the left substantia nigra and the left putamen, and the right substantia nigra and the right cuneus/precuneus/superior occipital gyrus. In all cases, the connectivity between these structures was significantly different in patients with RBD compared to both the Parkinson’s disease and healthy control groups. However, there was not always a difference between Parkinson’s disease and healthy controls, creating uncertainty on the relationship between these seed-based measures and nigrofugal pathway dysfunction. In contrast, our study used a data-driven approach to investigate the basal ganglia functional network as a whole. Unlike the previous seed-based study, we found no significant BGN differences between the RBD and Parkinson’s disease groups, whether comparing the groups on a voxel-wise or region of interest basis.

The apparent floor effect we have observed across the basal ganglia functional network in Parkinson’s disease and RBD subjects compared to the intermediate dopaminergic phenotype seen in RBD with ^123^I-ioflupane uptake may be best understood by appreciating what these different imaging modalities measure. Resting state functional MRI uses resting blood oxygen level-dependent signal to identify brain regions showing a strong temporal coherence (coactivation) in low frequency fluctuations (typically <0.1 Hz). These regions are defined as resting state networks, and reflect the intrinsic properties of brain organization (Filippini *et al.*, 2009). Increased default mode network (DMN) coactivation in *APOE* ε4 carriers at higher risk of future dementia has been demonstrated decades before any clinical, structural or neurophysiological correlate of neurodegeneration in young healthy adult carriers (Filippini *et al.*, 2009). These changes were unexplained by differences in memory performance, brain morphology or resting cerebral blood flow.

In our prodromal RBD subjects, the observed changes in BGN connectivity may occur years or even decades before the onset of clinical RBD symptoms, let alone significant motor impairment leading to a Parkinson’s diagnosis. Furthermore, it is well recognized that RBD subjects frequently manifest subtle features of motor impairment prior to their Parkinson’s disease diagnosis, supported by our finding of a mean motor UPDRS III score of 3.3 in the RBD cohort. This might suggest that RBD and Parkinson’s disease are not discrete clinical entities, but in fact manifestations of the same condition at different time points, with a detectable resting state functional MRI correlate very early in the disease evolution. Although longitudinal clinical and neuroimaging follow-up of the study groups is currently underway to formally assess this, our results would suggest that there is no increase in desynchronization within the BGN as individuals move from the premotor to the motor stage of Parkinson’s disease. This is consistent with our previous findings that basal ganglia connectivity does not correlate with the severity of motor impairment in established Parkinson’s disease (Szewczyk-Krolikowski *et al.*, 2014*a*; Rolinski *et al.*, 2015).

In contrast, dopaminergic function estimated with ^123^I-ioflupane SPECT or ^18^F-Fluorodopa PET is directly related to proportion of surviving substantia nigra dopaminergic neurons and related dopaminergic nerve terminal density, with its strongest clinical correlate being contralateral rigidity and bradykinesia (Leenders *et al.*, 1990). Our finding of an intermediate dopaminergic phenotype in RBD compared to Parkinson’s disease may simply reflect the relative temporal progression seen with these imaging modalities, with functional coherence being affected many years prior to the onset of dopaminergic neuronal degeneration. Furthermore, significant motor symptoms generally emerge only after 50–70% of dopaminergic nerve terminals have been irreversibly lost (Fearnley and Lees 1991), while compensatory or reactive changes in functional brain networks measured with resting state functional MRI will inevitably predate this by several years. Longitudinal studies will also help address the interesting question of whether the transition from RBD to Parkinson’s disease might be marked by changes in the functional coherence of resting state networks other than the BGN, such as the default mode network, which may be of particular relevance given the higher cognitive burden when early Parkinson’s disease is associated with concomitant RBD (Rolinski *et al.*, 2014).

We also detected reduced connectivity outside the basal ganglia, including a number of frontal areas, such as the cingulate, paracingulate and middle frontal gyri in Parkinson’s disease and RBD subjects compared to controls.

Functional connections between the basal ganglia and these frontal areas are known to be associated with executive function (Gordon *et al.*, 2015). Although executive dysfunction was not formally assessed in this study, global measures of cognitive function (Montreal Cognitive Assessment, Mini-Mental State Examination) and verbal fluency were reduced in RBD compared to controls, and in RBD compared to Parkinson’s disease subjects (Table 1). Interestingly, voxel-wise comparison did not reveal any statistically significant differences in these frontal areas when patients with RBD were compared to patients with established Parkinson’s disease, despite the observed clinical differences in global cognition and verbal fluency. Executive dysfunction is known to be common in early Parkinson’s disease (Dirnberger and Jahanshahi, 2013) and has also been shown to be associated with RBD (Massicotte-Marquez *et al.*, 2008). Our imaging findings would support this work.

Connectivity within the basal ganglia network differentiated patients with RBD from healthy controls with a sensitivity and specificity of 96.2% and 78.3%, respectively. While useful in itself, the greatest utility for this approach would be to facilitate the diagnosis of prodromal Parkinson’s disease, expressed as BGN network dysfunction in these subjects. However, the utility of BGN dysfunction as an imaging marker for the detection of prodromal Parkinson’s disease will only be addressed through careful longitudinal assessment of a larger RBD cohort, which is currently underway. We did not detect a significant correlation between BGN dysfunction and radiotracer uptake in the seven participants in whom both data were available, which may simply reflect a lack of statistical power. Despite best efforts, we were unable to perform SPECT scans in a larger RBD subgroup within the time constraints for this study, as participants were frequently unwilling to travel the longer distances incurred.

A previous longitudinal study with serial ^123^I-FP-CIT SPECT revealed significant decline in tracer uptake in patients with RBD, consistent with progressive nigrostriatal dopaminergic dysfunction (Iranzo *et al.*, 2011). Importantly, it was those patients with the lowest tracer uptake at baseline that developed Parkinson’s disease within the 3-year follow-up period. However, these results hold on a group level only, and due to considerable overlap of uptake values between RBD and controls, the predictive value of a single SPECT scan is limited. In contrast, resting state functional MRI analysis of BGN network dysfunction in our study yielded a sensitivity of 96.2% and specificity of 78.3%, indicating its potential as an indicator of early basal ganglia dysfunction. Moreover, compared to radiotracer imaging, resting state functional MRI does not carry an ionizing radiation burden; it is also cheaper and more readily accessible.

The advanced imaging techniques included in this study are currently research tools. Further independent validation and correlation with clinical outcomes will be necessary before they may be considered for true diagnostic use. Longitudinal clinical and MRI follow-up of our cohort, as well as acquisition of locally-acquired SPECT data, are currently underway to allow us to assess the potential for resting state functional MRI to predict the onset of Parkinson’s disease, and to investigate its relationship with dopaminergic dysfunction.

In our study, voxel-based morphometry analysis did not yield any significant grey matter differences between the three groups, including within cortex or the brainstem subregions, which could account for the differences in functional connectivity. Whilst previous studies have reported grey matter abnormalities associated with RBD (Ellmore *et al.*, 2010; Scherfler *et al.*, 2011; Hanyu *et al.*, 2012), subjects in these studies have generally had a longer reported RBD disease duration (9.2 years, Scherfler *et al.*, 2011) than the mean of 2.4 years in our relatively early cohort, which may have influenced results. Our findings mirror those in early Parkinson’s disease, where the use of structural compared to functional imaging has been somewhat disappointing (Menke *et al.*, 2014; Szewczyk-Krolikowski *et al.*, 2014*a*). One could therefore speculate that on the basis of our results, the imaging correlate of RBD progression to established motoric Parkinson’s disease is the evolution from functional network reorganization, through mild cortical and subcortical atrophy, followed by significant midbrain dopaminergic cell loss.

The diagnosis of RBD was confirmed through stringent clinical and polysomnographic assessment, but logistical and technical constraints meant that, in control subjects, the presence of RBD could not be formally excluded using polysomnography. However, the prevalence of RBD in the general population is low (Kang *et al.*, 2013), and accidental inclusion of such a subject would not impact negatively on our conclusions.

In conclusion, we have demonstrated resting state functional changes in the BGN of patients with RBD, and they mirror those of established Parkinson’s disease. Our findings support the presence of early basal ganglia dysfunction in these patients even before the onset of clinically relevant motor symptoms. Clinical and neuroimaging follow-up is necessary to assess the clinical utility of resting state functional MRI as an imaging biomarker to identify those most at risk of future conversion to the motor stages of Parkinson’s disease. This emerging MRI technique has the potential to deliver individualized risk assessment using a multimodal approach combined with other clinical measures, and has important implications for future neuroprotective trials in this key prodromal group.

## Abbreviations

BGN: basal ganglia network
RBD: REM sleep behaviour disorder
REM: rapid eye movement
SPECT: single photon emission computerized tomography
UPDRS: Unified Parkinson’s Disease Rating Scale
